# Predicting language outcomes after stroke: Is structural disconnection a useful predictor?

**DOI:** 10.1016/j.nicl.2018.03.037

**Published:** 2018-03-30

**Authors:** Thomas M.H. Hope, Alex P. Leff, Cathy J. Price

**Affiliations:** a Wellcome Centre for Human Neuroimaging, University College London, UK; b Institute of Cognitive Neuroscience, University College London, UK; c Department of Brain, Repair and Rehabilitation, Institute of Neurology, University College London, UK

**Keywords:** Stroke, Language, Aphasia, Outcomes, MRI, Connectomics, White matter

## Abstract

For many years, researchers have sought to understand whether and when stroke survivors with acquired language impairment (aphasia) will recover. There is broad agreement that lesion location information should play some role in these predictions, but still no consensus on the best or right way to encode that information. Here, we address the emerging emphasis on the structural connectome in this work – specifically the claim that disrupted white matter connectivity conveys important, unique prognostic information for stroke survivors with aphasia.

Our sample included 818 stroke patients extracted from the PLORAS database, which associates structural MRI from stroke patients with language assessment scores from the Comprehensive Aphasia Test (CAT) and basic demographic. Patients were excluded when their lesions were too diffuse or small (<1 cm3) to be detected by the Automatic Lesion Identification toolbox, which we used to encode patients' lesions as binary lesion images in standard space. Lesions were encoded using the 116 regions defined by the Automatic Anatomical Labelling atlas. We examined prognostic models driven by both “lesion load” in these regions (i.e. the proportion of each region destroyed by each patient's lesion), and by the disconnection of the white matter connections between them which was calculated via the Network Modification toolbox. Using these data, we build a series of prognostic models to predict first one (“naming”), and then all of the language scores defined by the CAT.

We found no consistent evidence that connectivity disruption data in these models improved our ability to predict any language score. This may be because the connectivity disruption variables are strongly correlated with the lesion load variables: correlations which we measure both between pairs of variables in their original form, and between principal components of both datasets. Our conclusion is that, while both types of structural brain data do convey useful, prognostic information in this domain, they also appear to convey largely the same variance. We conclude that connectivity disruption variables do not help us to predict patients' language skills more accurately than lesion location (load) data alone.

## Highlights

• Structural connectivity appears important to language and disorders of language
• We asked whether we could use connectivity variables to predict language outcomesafter stroke more accurately
• We found no consistent evidence of any predictive advantage for any language outcome

## 1 Introduction

For many years, researchers have tried to understand and predict whether and when stroke survivors will recover lost speech and language abilities (Bang et al., 2005; Cloutman et al., 2009; Crinion and Price, 2005; Hope et al., 2017; Hope et al., 2013; Konig et al., 2008; Lazar et al., 2008; Lendrem and Lincoln, 1985; Marshall and Phillips, 1983; Payabvash et al., 2010; Pedersen et al., 1995; Seghier et al., 2016; Tilling et al., 2001; Ween et al., 2000). There is broad agreement that lesion location information should play some role in this work (Plowman et al., 2012), but still no consensus on the best or right way to encode that information (Forkel et al., 2014; Hope et al., 2013; Mah et al., 2014; Price et al., 2017; Zhang et al., 2014). An emerging emphasis on structural (i.e. white matter) connectivity in studies of language has naturally encouraged the same attention in studies of aphasia (Agosta et al., 2010; Epelbaum et al., 2008; Fridriksson et al., 2013; Hope et al., 2016; Olsen et al., 2015; Ripamonti et al., 2014). As many studies have shown that disrupted connectivity contributes to language impairments and their recovery (Forkel et al., 2014; Hope et al., 2016; Kuceyeski et al., 2015a; Pani et al., 2016; Wu et al., 2015; Yourganov et al., 2016), it is natural to presume that connectivity disruption data should be pivotal when predicting language outcomes after stroke.

However, lesion distributions are highly structured (Inoue et al., 2014; Mah et al., 2014). If one brain region is damaged, neighbouring regions are often damaged too, and white matter disruption will tend to be highly correlated with cortical damage. So even if connectivity disruption is the causal mechanism for some post-stroke cognitive symptoms, it may be that lesion location can serve as a reliable proxy in prognostic models. We might find that the addition of connectivity disruption data adds little, unique prognostic value to our models of post-stroke outcomes. Or to put the point another way, mechanistic importance is no guarantee of clinical importance, in this domain. In what follows, we test the clinical importance of connectivity disruption data in a very large sample stroke patients.

## 2 Methods

### 2.1 Patient data

Our patient data were extracted from our *PLORAS* database (Seghier et al., 2016), which associates stroke patients, tested over a broad range of times post stroke, with demographic data, behavioural test scores from the Comprehensive Aphasia Test (Swinburn et al., 2004), and high resolution T1-weighted MRI brain scans. Patients are excluded from the PLORAS database when there is evidence they have other neurological conditions (e.g. dementia, multiple sclerosis), contraindications to MRI scanning, are unable to see or hear the stimuli required to assess their language abilities, or have insufficient comprehension of the purpose of the study to provide consent for their participation. We included all patients whose data was available at the time, irrespective of their: age at stroke onset; sex; premorbid handedness; or native language. Patients were only excluded if their lesions were too diffuse or small (< 1cm^3^) to be detected by our Automatic Lesion Identification (ALI) toolbox (Seghier et al., 2008).

### 2.2 Structural brain imaging data

Imaging data were collected using sequences described elsewhere (Hope et al., 2015). Data from different scanners were combined after conversion to quantitative probabilistic estimates of grey matter density. Pre-processed with Statistical Parametric Mapping software (SPM, 2012), these images were spatially normalised into Montreal Neurological Institute (MNI) space using a modified version of the unified segmentation algorithm (Ashburner and Friston, 2005) that has been optimized for use in patients with focal brain lesions (Seghier et al., 2008). We used the ALI toolbox (Seghier et al., 2008) to index the degree of abnormality at each voxel in each patient image (in relation to the same type of images in healthy controls), combining the grey and white matter outputs to generate a single thresholded (i.e. binary) image that shows the presence or absence of a lesion at each voxel. Lesion volume is calculated as the sum of those voxels where lesions were deemed to be present.

Following the approach taken by Yourganov and colleagues (Yourganov et al., 2016), in a recent study which demonstrates that connectivity disruption data can drive useful predictions for language outcomes after stroke, we encoded our lesion images using the 116 grey-matter regions defined by the Automatic Anatomical Labelling atlas (Tzourio-Mazoyer et al., 2002a). We examined models driven by both lesion load in these regions (i.e. the proportion of each region destroyed by each patient's lesion), and by the disconnection of the white matter connections between them. Disconnection was calculated via the Network Modification toolbox (Kuceyeski et al., 2013), which generates the mean disconnection implied by each lesion, using structural connectomes defined for a separate sample of 73 neurologically normal controls. This toolbox has been used to successfully predict both network atrophy (Kuceyeski et al., 2014) and cognitive outcomes (Kuceyeski et al., 2016) after stroke, and has also been successfully employed in studies of longitudinal patterns of atrophy in Alzheimer's patients (Raj et al., 2015), the spread of Progressive Supranuclear Palsy(Pandya et al., 2017), cortical atrophy in temporal lobe epilepsy (Abdelnour et al., 2015), and early Multiple Sclerosis (Kuceyeski et al., 2015b).

### 2.3 Behavioural data

Every patient was assessed using the Comprehensive Aphasia Test (CAT) (Swinburn et al., 2004). For ease of comparison across tasks, task scores are expressed as T-scores, representing each patient's assessed skill on each task (e.g., describing a picture; reading non-words) relative to a reference population of 113 aphasic patients. The threshold for impairment is defined relative to a separate population of 27 neurologically normal controls such that performance below threshold would place the patient in the bottom 5% of the normal population (Swinburn et al., 2004). Lower scores indicate poorer performance. The CAT yields 34 separate scores, though six refer to non-linguistic skills such as line bisection, arithmetic and memory. Here, we focus initially on scores in naming (i.e. of visually presented pictures), before widening the analysis to include all of the other 27 language scores. Detailed descriptions of the tasks are given in the CAT manual (Swinburn et al., 2004).

### 2.4 The baseline model

Our aim here was to measure what the introduction of structural (dis)connection variables buys us, in terms of improved predictive accuracy. Our baseline for this comparison, is a model driven by variables whose prognostic relevance is already supported by prior evidence: (i) basic demographic data including time post-stroke (Hope et al., 2017; Hope et al., 2013), age at stroke (Ramsey et al., 2017), pre-stroke handedness (Knecht et al., 2000), and bilingualism (Hope et al., 2015); (ii) lesion volume (Plowman et al., 2012); and (iii) lesion location (Hope et al., 2013; Plowman et al., 2012; Yourganov et al., 2016), which is calculated as described above. We use the term ‘lesion load variables’ to refer to variables representing the proportion of each of a series of anatomically defined regions, which is destroyed or encroached upon by each patient's lesion(s). We use the term ‘lesion load model’ to refer to models driven by the combination of: (a) demographic and lesion volume variables, as described above; and (b) lesion load variables.

### 2.5 Structural connectivity models

To measure whether structural connectivity variables add prognostic information over and above that already conveyed by lesion-load models, we compare the predictions made by lesion-load models to those made using models which either: (a) replace the lesion load variables with structural connectivity variables, or (b) add structural connectivity variables to the lesion-load model, or (c) stack lesion-load and connectivity models together. Like the lesion load model, all of these models also include basic demographic data and lesion volume. For the sake of brevity, we refer to them as: “connectivity models”, ‘lesion load plus connectivity models’, and “stacked models” in what follows.

Stacking starts by training component models separately (e.g. a lesion-load model and a structural connectivity model), and using those models to predict the language scores under study via cross-validation. The resulting predictions are then used as input to a new model, also trained to predict the same language scores. This new, higher level model is also assessed in cross-validation, using the same folds as employed to generate the predictions from the component models. Our use of this approach is motivated by recent work which employs stacking to apparently good effect in this domain (Pustina et al., 2017), reporting modest but significant improvements in predictive power over what was possible with any component model alone. More generally, stacking is thought to be useful when – as here – we want to combine inferences made from datasets containing very unequal numbers of variables. The argument is that if such datasets are merely appended, the larger set may dominate the resulting model, even at the cost of increased prediction error (Pustina et al., 2017).

### 2.6 Feature selection

Following the approach recently preferred by Yourganov and colleagues (Yourganov et al., 2016), we applied an initial, mass univariate filter to the lesion load and connectivity variables in all of our models. Correlating each variable, singly, with our target language score, we kept only those variables for which the result was significant after a Bonferroni correction for multiple comparisons. This is a convenient if not particularly powerful feature selection method, but alternatives such as wrapper selection (Kohavi and John, 1997; Pustina et al., 2017), in which features are added or removed sequentially based on more direct measures of their predictive utility, are also prone to overfitting, which can dramatically reduce out-of-sample predictive performance (Pustina et al., 2017).

Surprisingly, our feature selection filter left more than a thousand connectivity disruption variables included when regressed against many language scores. Models with too many variables are known to suffer from a ‘curse of dimensionality’, which hampers their predictive power; left as it was, we thought that the comparison between the lesion load and connectivity models would not be fair (biased in favour of lesion load). So we repeated each analysis using a second restricted set of connectivity variables that were equal in number to the load variables: i.e. if the filter led to the selection of N lesion load variables when predicting a given language score, we selected the N connectivity variables with the strongest correlations to the same language score.

### 2.7 Model comparison

Model performance was assessed via 10 times 10-fold cross-validation (Kohavi and John, 1997), with the same folds used for every analysis focused on predicting the same language score. Feature selection, as described above, was performed within each fold, using only the training data for that fold. This process yields 10 predictions per patient, and the final prediction is their mean average. One intuitive way to measure the quality of these predictions, is via the coefficient of the correlation between predicted and empirical scores. Larger coefficients imply better predictions (Hope et al., 2015; Pustina et al., 2017; Yourganov et al., 2016), and the coefficients can be compared directly via a Fischer r-to-z transform (Pustina et al., 2017). However, while usually related, these correlation coefficients are also potentially orthogonal to prediction error: for example, the correlation between predicted and empirical scores is unaffected if we add a constant to all predictions, whereas this manipulation will certainly affect prediction error. For this reason, we compare models by comparing their prediction errors directly: specifically, by comparing the variances of their prediction error distributions: more accurate predictions have smaller prediction error distribution variances. But we also use correlation coefficients as a convenient and intuitive way to report model quality.

Our analysis involves comparing the prediction error distribution variances of our lesion-load model to those of all of the connectivity models: i.e. (a) when lesion load variables are replaced by connectivity variables (i.e. producing a connectivity model); (b) when lesion-load and connectivity variables are appended (i.e. producing a lesion load plus connectivity model); and (c) when lesion load models are stacked with connectivity models (i.e. producing a stacked model). Each connectivity model is reproduced twice, with either the full or the restricted connectivity datasets for each task analysis, so there are a total of 6 model comparisons to make. Each comparison is a one-tailed variance test, because we are only interested in situations where the prediction error distribution variances for the lesion load models are significantly larger (worse) than they are for any connectivity model.

### 2.8 Inducers

There are many different ways to tackle regression problems; here, we consider a range of popular alternatives. Our analysis begins with support vector machines, with a linear kernel, simply because this inducer has been more prominent than others in the recent, relevant literature: e.g. (Mah et al., 2014; Yourganov et al., 2016). But we go on to employ all of the different inducers distributed in the Matlab 2017a Classification Learner application: (i) multiple linear regression; support vector machines with (ii) linear; (iii) quadratic; (iv) polynomial order 3; and Gaussian kernels with kernel scales of (v) 5; (vi) 10; and (vii) 20; Gaussian Process regression models with (viii) squared exponential; (ix) rational quadratic; (x) exponential; and (xi) matern5/2 kernel functions; regression trees with minimum leaf sizes: (xii) 4, (xiii) 12 and (xiv) 36; and (xv) boosted and (xvi) bagged regression trees. Taken together, these methods represent a reasonable cross-section of the current state of the art in regression modeling.

### 2.9 Omnibus analysis

With 16 inducer configurations, 28 language scores from the CAT (Swinburn et al., 2004), and 7 model predictor configurations to test, we had a total of 3136 repetitions of the core (10 × 10-fold) cross-validation process to complete (112 per task analysis). These were run with Matlab 2017b, on a 16-core PC running Windows 8.1, and took ~72 h to complete. Full results are available in Supplementary Material: here, we summarise the key features of those results.

## 3 Results

### 3.1 Lesion and language data

**Fig. 1:**
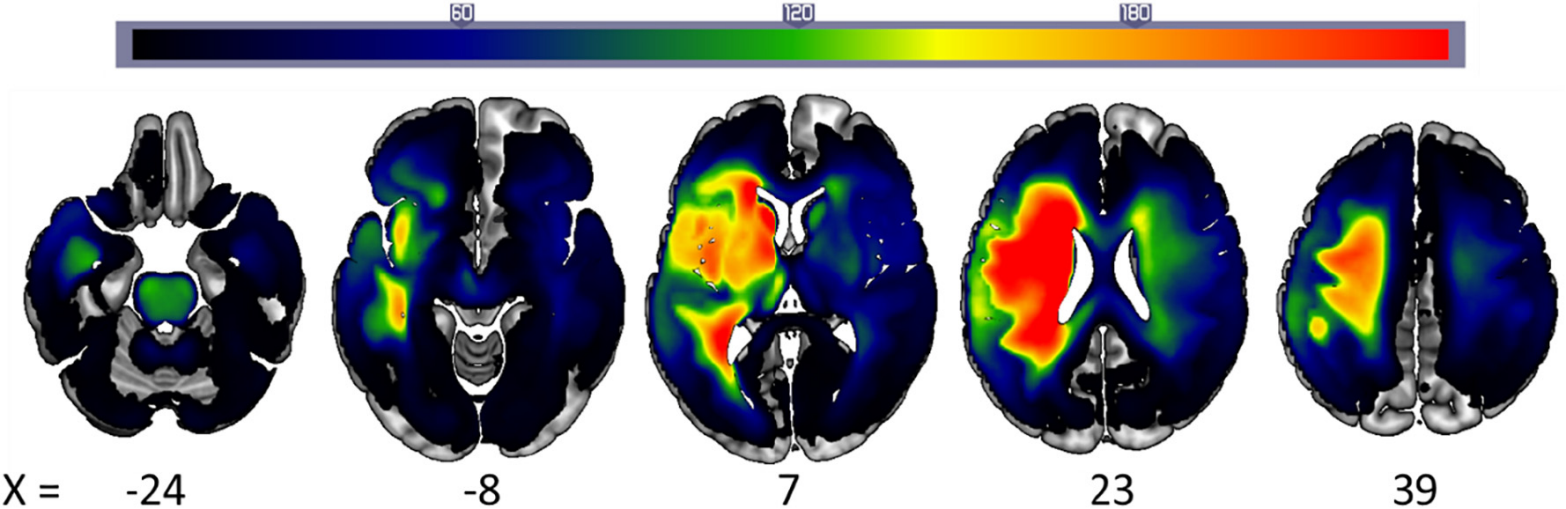
Lesion frequency. Axial slices of a lesion frequency image for 818 patients.

There were a total of 818 patients in our sample, including 260 women and 98 patients who were left-handed or ambidextrous pre-stroke. Their mean age at stroke onset was 55 years (standard deviation = 13 years), and the mean time after stroke onset at which they were assessed was 58 months (standard deviation = 66 months). Fig. 1 illustrates the distribution of the patients' lesions. Median scores and ranges for each task score are included in Table 1.

**Table 1:** Predictive performances (simple correlations between predicted and empirical = Pearson's R) of the best of 16 inducers for each language score and data configuration. No model which employed connectivity variables was significantly better than the lesion load model, when predicting any language score (all *p* > 0.2). Med. = median; N = sample size; L = lesion load model; C(r) = restricted connectivity model; C(f) = full connectivity model; LC(r) = lesion load appended to restricted connectivity; LC(f) = lesion load appended to full connectivity; LsC(r) = stacked model with lesion load and restricted connectivity; LsC(f) = stacked model with lesion load and full connectivity.

TASK			R: Predicted vs. Empirical						
	Med. (range)	N (all/impaired)	L	C(r)	C(f)	LC(r)	LC(f)	LsC(r)	LsC(f)
Fluency	68 (38)	812/255	0.72	0.73	0.73	0.73	0.73	0.72	0.70
Comprehension of spoken words	65 (40)	814/158	0.50	0.51	0.50	0.51	0.50	0.51	0.50
Comprehension of spoken sentences	63 (44)	813/370	0.66	0.67	0.66	0.67	0.66	0.67	0.65
Comprehension of spoken paragraphs	60 (26)	805/116	0.44	0.43	0.39	0.43	0.40	0.44	0.45
Comprehension of spoken language	63 (48)	805/283	0.66	0.67	0.66	0.67	0.67	0.66	0.65
Comprehension of written words	65 (37)	813/256	0.54	0.53	0.55	0.54	0.55	0.54	0.53
Comprehension of written sentences	64 (47)	809/278	0.67	0.66	0.66	0.68	0.67	0.66	0.64
Comprehension of writing	65 (48)	808/339	0.67	0.66	0.67	0.68	0.67	0.67	0.65
Repeating words	57 (30)	813/312	0.63	0.64	0.65	0.63	0.64	0.63	0.62
Repeating complex words	62 (24)	812/252	0.64	0.66	0.64	0.65	0.64	0.64	0.62
Repeating non-words	67 (29)	813/233	0.57	0.57	0.56	0.57	0.56	0.57	0.58
Repeating digit strings	66 (31)	815/253	0.70	0.70	0.69	0.69	0.69	0.70	0.70
Repeating sentences	63 (24)	811/293	0.76	0.75	0.75	0.76	0.75	0.76	0.75
Repeating (all)	58 (38)	810/445	0.73	0.74	0.74	0.74	0.74	0.74	0.73
Object naming	66 (37)	815/352	0.71	0.72	0.72	0.72	0.72	0.72	0.70
Action naming	69 (30)	813/420	0.68	0.70	0.69	0.70	0.69	0.70	0.68
Naming (all)	69 (40)	807/341	0.74	0.75	0.75	0.75	0.75	0.75	0.74
Spoken picture description	63 (36)	805/397	0.72	0.73	0.73	0.73	0.73	0.73	0.72
Reading words	69 (31)	809/362	0.68	0.70	0.69	0.69	0.69	0.68	0.67
Reading complex words	67 (27)	805/304	0.69	0.69	0.69	0.69	0.69	0.69	0.68
Reading function words	62 (27)	808/97	0.60	0.60	0.60	0.60	0.60	0.58	0.58
Reading non-words	61 (28)	807/330	0.70	0.70	0.70	0.70	0.69	0.70	0.69
Reading	66 (33)	805/335	0.72	0.73	0.73	0.73	0.73	0.72	0.71
Writing (copying)	61 (28)	796/101	0.45	0.43	0.43	0.44	0.43	0.38	0.38
Written picture naming	67 (29)	801/189	0.58	0.60	0.59	0.59	0.58	0.58	0.56
Writing to dictation	68 (30)	799/299	0.68	0.68	0.67	0.68	0.67	0.69	0.68
Writing	65 (35)	786/270	0.67	0.67	0.66	0.67	0.67	0.69	0.67
Written picture description	71 (33)	781/354	0.71	0.71	0.71	0.72	0.71	0.71	0.71

### 3.2 Analysis 1: naming

**Fig. 2:**
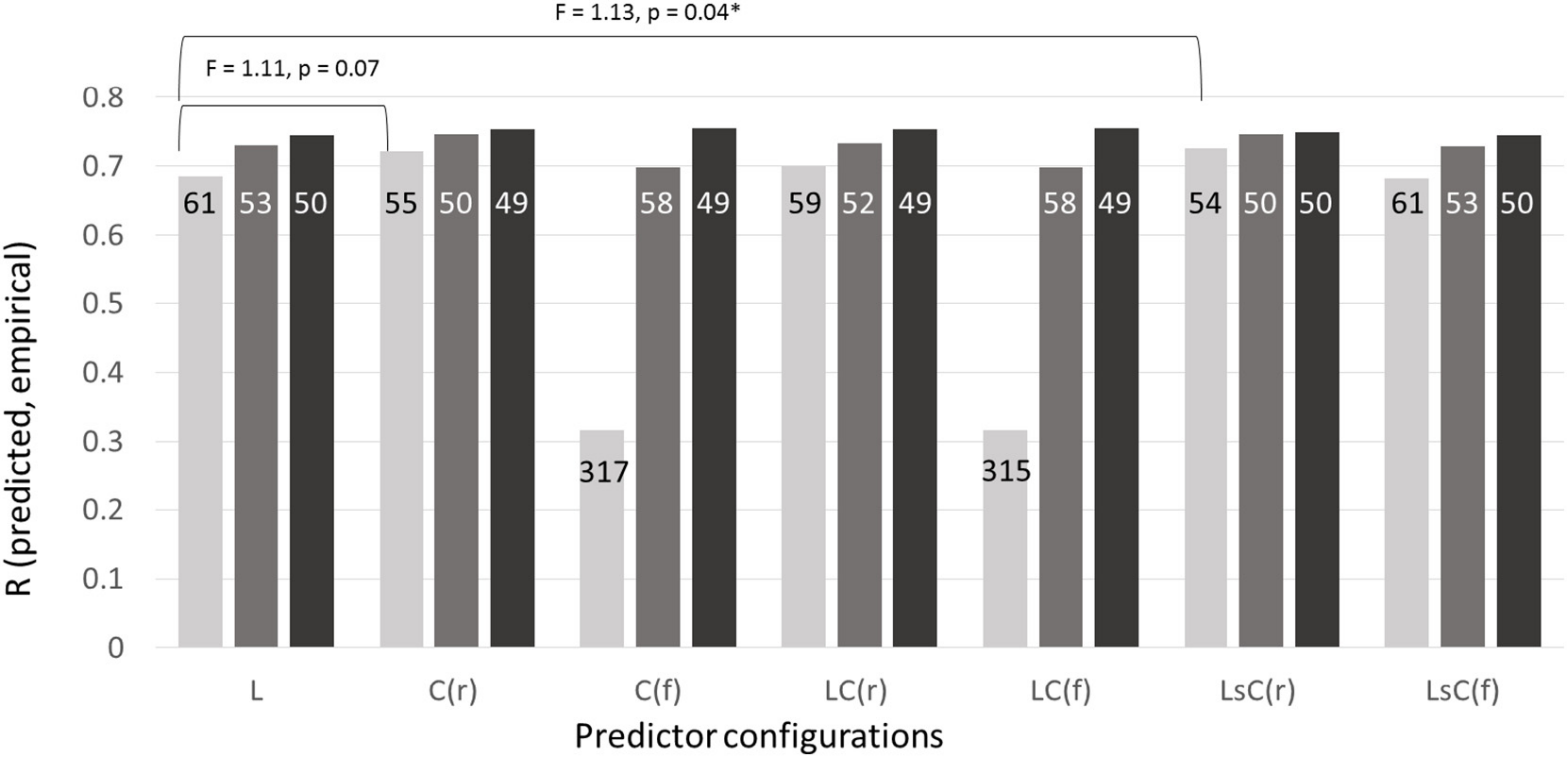
Predictive performance on naming scores. Model predictive performance is shown for: (a) linear support vector machines (light grey bars); (b) Gaussian processes with a rational quadratic kernel (mid-grey bars); and (c) the best of 16 inducers tried (dark grey bars). Models were trained on each of 7 data configurations: (i) lesion load only, L; (ii) restricted connectivity disruption, C(r); (iii) full connectivity disruption, C(f); (iv) lesion load plus restricted connectivity, LC(r); (v) lesion load plus full connectivity, LC(f); (vi) lesion load stacked with restricted connectivity, LsC(r); and, (vii) lesion load stacked with full connectivity, LsC(f). When produced using a linear support vector machine, there was a marginally significant benefit for the stacked model using lesion load and restricted connectivity (*p* = 0.04), and non-significant trend for the model which simply replaced lesion load with restricted connectivity (*p* = 0.07). No significant benefits were observed when predictions were made using either GPMR (all p > 0.1) or the best of 16 inducers (all p > 0.2). Numbers in each bar are prediction error distribution variances: all of the model comparisons are comparisons of these variances.

To make the structure of the analysis as clear as possible, we start by reporting results from a single inducer, employed to predict patients' scores in a single language skill: naming. Naming is a popular focus for research in aphasia because deficits of this skill, anomia, are perhaps the most common of the persistent language impairments that stroke survivors suffer. We began the analysis using a support vector machine with a linear kernel, simply because this is the most popular inducer both in our field (e.g. (Mah et al., 2014; Yourganov et al., 2016)) and in other studies which aim to use structural neuroimaging to predict labels of clinical interest (Arbabshirani et al., 2017). With this inducer, we can see some evidence both that the intuition behind restricting the connectivity disruption data was right, because the restricted connectivity models tend to perform at least as well as the full connectivity models, and that the use of that data significantly improves our ability to predict the patients' naming skills (see Fig. 2).

When tackling these prediction problems in the past, we have preferred Gaussian process model regression with a rational quadratic kernel (e.g. (Hope et al., 2013)) to support vector regression with linear kernel. Linear inducers are often preferable because their weights are easier to interpret, but our impression is that non-linear inducers are more powerful in this domain. This impression was confirmed when we repeated the previous analysis with our preferred inducer: the results with all predictor configurations are better, and in particular the Gaussian process models appear to handle the full connectivity datasets more gracefully (see Fig. 2). But critically, this inducer also extinguished any apparent benefit of using connectivity disruption data to predict naming scores (see Fig. 2; all *p* > 0.1).

In effect, we have opened a Pandora's box here, because our comparisons of interest appear to be inducer-dependent. This is confirmed when the same analysis is repeated with many different inducers (as listed in the Methods): analyses with 5/16 inducers reveal what seem to be significant benefits of the use of the structural connectivity data (though only 3/16 would survive a correction for multiple comparisons), but there are no significant benefits when we use the other 11 inducers. When we take just the best result (i.e. with the smallest prediction error distribution variance) for each configuration of predictors, the results are consistent and there are no significant differences (all *p* > 0.3; see Fig. 2). In other words, when we are as sure as we reasonably can be that we are making best use of the available predictors, structural connectivity variables do not drive significantly better predictions of naming scores.

### 3.3 Predicting all the language scores

We next turned to all of the other 27 language scores defined by the CAT: repeating all of the analyses described so far for every one of those scores. Since our comparisons of interest were inducer dependent in the last section, we now report only those comparisons of predictions derived from the best inducer for each predictor configuration (Table 1). The sample sizes vary across language scores, because some patients had missing data in some tasks, but even the minimum sample size is very large (781). The results of these analyses are all essentially similar to those that we found for naming: we find no evidence that the use of connectivity disruption data significantly improves our ability to predict any language score (all *p* > 0.2).

### 3.4 Connectivity disruption is correlated with lesion load

Finally, we sought to understand why the prior analyses yield no significant benefits, by comparing the lesion load and connectivity disruption variables to each other. Specifically, we hypothesised that both data types convey a great deal of shared information, and this does appear to be true. First, we identified all of the lesion load and connectivity disruption variables which could reasonably be correlated: i.e. those that were affected by at least 3 patients' lesions. All of the 116 lesion load variables met this criterion, but only 2420 connectivity variables were included. Pairwise correlations between these variables revealed that every lesion load variable was correlated with at least one connectivity variable and vice versa, even after a Bonferroni correction for multiple comparisons. These two sets of variables are highly correlated.

**Fig. 3:**
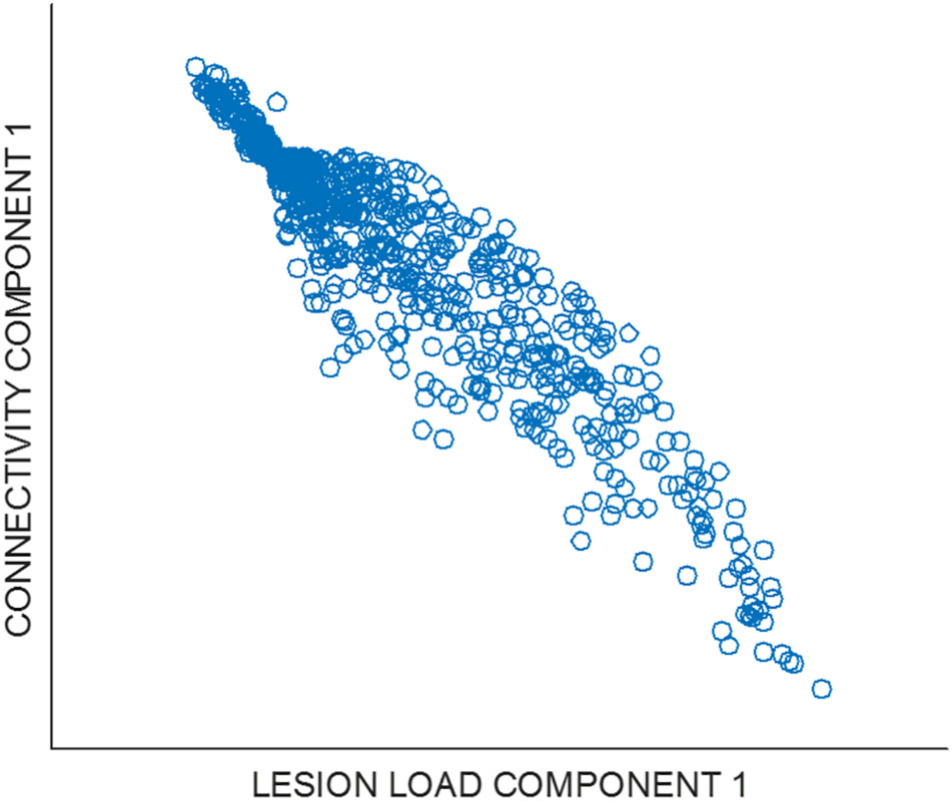
Scatter plot of the first principal component of the lesion load data versus the first principal component of the connectivity disruption data.

Another way to make this measurement is by reducing the dimensionality of each dataset separately, and measuring the correlations between the principle components of each set. Here again, and having also applied a Bonferroni correction for multiple comparisons, significant pairwise correlations existed for every principle component which, individually, explained at least 1% of the total variance in the original data (11 components of the lesion load variables, and 13 components of the connectivity variables). Fig. 3 presents an example of this correspondence, plotting the first principal components of each dataset against each other (*r* = −0.94).

## 4 Discussion

We found no evidence that the addition of connectivity disruption data improves our ability to relate the lesions a patient has suffered to any subsequent language outcome. When predicting language outcomes after stroke, we found no use for connectivity disruption data, over and above what we could garner from lesion location (load) data.

One caveat to these results is that we have inferred connectivity disruption from T1-weighted MRI, rather than measuring it “directly” with subject-specific diffusion data. Results derived from the Network Modification toolbox, which we used to make those inferences, have been validated elsewhere (Kuceyeski et al., 2013; Kuceyeski et al., 2015a), but subject-specific information is still lost here. Quite how important this is remains to be seen. Two recent studies from the same lab (Del Gaizo et al., 2017; Yourganov et al., 2016) used subject-specific diffusion data to run similar analyses to those that we employed here, albeit with smaller samples and a single inducer: a linear support vector machine. The earlier study (Yourganov et al., 2016) reported apparent advantages when using their connectivity data, but did not report a formal model comparison to quantify that benefit. The later study (Del Gaizo et al., 2017) does report a formal model comparison, but finds no significant predictive benefit of including their connectivity data. In this respect, our results are actually more positive than theirs, because we find significant advantages in 9/28 tasks when using the same inducer (a linear support vector machine), albeit only without a correction for multiple comparisons (see Supplementary Material). The only study that we know of which does report a formally quantified advantage over and above lesion load, is that by Pustina and colleagues (Pustina et al., 2017); these authors inferred their connectivity variables much as we have done, rather than using subject-specific diffusion data. So at present, there is no evidence that subject-specific diffusion data makes a critical difference here.

Superficially, our results appear to directly contravene those reported by Pustina and colleagues (Pustina et al., 2017); they reported a significant advantage over lesion load only models, where we found no such advantage. Notably, their analysis also employed variables derived from resting state fMRI, and did not distinguish whether the advantages they saw were driven by the connectivity data, the fMRI, or both. Our results and theirs could be compatible, in other words, if the key driver of the advantage they report is the fMRI data. However, these authors also quantified that advantage using paired *t*-tests to compare sets of correlation coefficients (predicted versus empirical task scores) generated from 20 repetitions of a 10-fold cross-validation process. This is a considerably more permissive test than we have used here. As an illustration, the lesion load models in our analyses rarely have the highest correlation coefficients in Table 1: a paired t-test reveals that coefficients for the lesion load plus restricted connectivity (LC(r)) models are significantly higher across all language tasks, than those for the lesion load (only) models (*t* = 3.95, *p* = 0.001). But the mean improvement in correlation coefficients here is just 0.005: numerical differences this small can hardly make a compelling case for the use of connectivity data.

Another caveat flows from the method we used to segment the patients' lesions. Most prior studies in this area employ binary lesion images to predict stroke patients' language scores (Del Gaizo et al., 2017; Hope et al., 2015; Hope et al., 2016; Mah et al., 2014; Ramsey et al., 2017; Yourganov et al., 2016), so we used the same approach to maximise the comparability between our study and that earlier work. Nevertheless, several recent studies have suggested that algorithmic approaches to binary lesion segmentation should be treated with caution, at least in the sense that they may diverge from the presumed “gold standard” of manual segmentation by a neurologist (Pustina et al., 2016; Yourganov et al., 2016). In response, we would emphasise that our lesion images were all checked, by eye, by experienced neuroscientists: this process should highlight any dramatic or systematic artefacts in these data. And in any case, our aim is to capture the information in lesion images which predicts cognitive/behavioural outcomes, rather than to maximise the similarity between manually and automatically segmented lesions. Our predictive results are at least comparable, and often favourably so, with those reported in other recent work (Del Gaizo et al., 2017; Ramsey et al., 2017; Yourganov et al., 2016; Zhang et al., 2014), which suggests that we are capturing most of the relevant variance in the patients' lesions.

A third caveat concerns the regions of interest used to encode both lesion load and connectivity disruption, regions derived from the Automatic Anatomic Labelling atlas (Tzourio-Mazoyer et al., 2002b). This was a pragmatic choice, made because: (a) comparable, recent studies have used this atlas (Del Gaizo et al., 2017; Yourganov et al., 2016); and (b) the Network Modification toolbox also works with the same atlas (Kuceyeski et al., 2015a). Our own experience is that different parcellations of the brain do not drive dramatically different predictive power in this domain, but it is certainly conceivable that an alternative parcellation might drive predictions which are both better than those reported here, and which also show more significant benefits associated with the use of connectivity disruption data. Indeed, even without a different parcellation, there are many different ways to represent connectivity disruption which we have not considered here, such as the dynamical measures recently employed to good effect by Del Gaizo and colleagues (Del Gaizo et al., 2017), though note this measure did not significantly improve on lesion load models either. Some alternative representation or encoding of this data may yet reveal a more significant role for connectivity-based analyses. The same logic applies more widely too: since our comparisons of interest are inducer-dependent, there is always the chance that some new inducer will be better than those used here, while also making a stronger case for the use of connectivity disruption data.

Even with those caveats in mind, we contend that these results should encourage caution over claims surrounding the clinical utility of structural connectivity data in this area. White matter connectivity is difficult to measure, particularly in the damaged brain, and the diffusion weighted MRI required to make those measurements precisely is far from routine in clinical care. Neither of those challenges is insuperable, if we can make a compelling case that this kind of analysis is really critical to post-stroke prognostics; however, our experiences so far, as reported here, suggest that this case may be rather more difficult to make than many might have hoped or expected it to be. In fact, this case might be even more difficult to make than our results suggest, because there are good reasons to suspect that our lesion-load models are not as powerful as they could be. For example, Rondina and colleagues (Rondina et al., 2016) recently showed that a voxel-level encoding of lesion location data drove significantly better predictions of outcomes for patients suffering from hemiparesis after stroke, than did a regional lesion load encoding like that used here. If an alternative encoding like this, which does not employ structural connectivity data, can improve on the predictive performance of our lesion load models in the language domain, the case against using structural connectivity variables will grow stronger.

We expected to find some benefit here, mainly because we imposed a wholly artificial limitation on the lesion-load-only models, by only including lesion load related to grey matter locations rather than including lesion load related to white matter tracts. This limitation is easy to circumvent by including white matter tracts as regions of interest – as we have done routinely in the past (Hope et al., 2015; Hope et al., 2013; Hope et al., 2016). Accordingly, we expected to find an initial benefit of using structural connectivity variables, which was then either reduced or eliminated when our baseline models were expanded to capture white matter lesion load. We never needed to take that extra step because the expected, initial advantage never emerged. This begs the question of how our models treat patients with white-matter-only lesions: in fact, there were only 7 patients with these lesions in our sample (which excluded lacuna infarcts that were smaller than 1cm^3^), and all had language scores in the normal range in most tasks. This might indicate that these patients are either rare, or rarely suffer the enduring language impairments which might encourage participation in a study like ours. Another explanation is that our sample does include patients with white matter only lesions, but that our grey matter regions are simply liberal, in the sense that they encroach into what a neurologist might call white matter, thereby capturing enough of the key lesion-symptom trends embodied by these patients to predict their language skills at least reasonably well.

In conclusion, we found that our ability to predict language outcomes after stroke was not significantly improved for models that included white matter connectivity disruption. Some sort of improvement is necessary to justify the claim that any neuroimaging data modality is clinically useful in this domain. We do not question the popular presumption that connectivity is important to language, nor that disconnection is important to impairments of language: we ourselves have recently shown that the latter is likely to be true (Hope et al., 2016). But our results here suggest that lesion load variables can serve as reliable proxies for connectivity disruption data in prognostic models. We hope that this result will encourage others to make similar analyses, establishing whether and how structural connectivity data can be used to reap the promised, predictive benefit.

## Author contributions

TMHH and CJP conceived the analyses, and TMHH implemented them. TMHH also led the writing of the manuscript, though all co-authors supported this process. CJP established the processes for acquiring the patient data, with support from APL, who helped curate it.

## Author Information

All authors declare that they have no competing financial interests in this work.

## Appendix A Supplementary data

Supplementary material: the omnibus analysis results in full. Image 1
